# Comparative Study of Raw Ham and Other Pork Tissues for the Detection of *Toxoplasma gondii*

**DOI:** 10.3390/foods14244350

**Published:** 2025-12-17

**Authors:** Daniel Berdejo, Laura Herrero, María Jesús Gracia, Ignacio de Blas, Regina Lázaro, Susana Bayarri

**Affiliations:** 1 Departamento de Producción Animal y Ciencia de los Alimentos, Facultad de Veterinaria, Instituto Agroalimentario de Aragón—IA2 (Universidad de Zaragoza—CITA), 50013 Zaragoza, Spain; berdejo@unizar.es (D.B.);; 2 Departamento de Patología Animal, Facultad de Veterinaria, Instituto Agroalimentario de Aragón—IA2 (Universidad de Zaragoza—CITA), 50013 Zaragoza, Spain; mjgracia@unizar.es (M.J.G.); deblas@unizar.es (I.d.B.)

**Keywords:** *Toxoplasma gondii*, raw ham, pork tissues, mouse bioassay, qPCR, meat industry

## Abstract

*Toxoplasma gondii* has long been a significant food safety concern, as standardized methods for detecting and controlling it are still lacking in the slaughterhouse and the broader meat industry. We evaluated the presence of *T. gondii* in raw ham and in target tissues (heart and tongue) of seropositive pigs with the aim of selecting a representative risk evaluation tissue to test raw pork destined for the elaboration of cured meat products. To increase the sensitivity of *T. gondii* detection, we additionally employed bioassay in mice and qPCR analysis. *T. gondii* was detected in 26 raw hams and in 26 samples of target tissues of the 38 seropositive pigs analyzed (68.4%). Statistical analysis demonstrated a strong agreement between the results obtained from the ham and target tissue samples (κ = 0.790). Moreover, the antibody titers obtained in mice inoculated with target tissue were higher (up to 1:320) compared with those inoculated with raw ham. These findings suggest that tissues such as tongue and heart, which have less commercial value than raw hams, can serve as primary selection tissues for the detection of *T. gondii*. Consequently, they could serve as a valuable and effective raw material control tool in the dry-cured ham industry.

## 1. Introduction

*Toxoplasma gondii* is a protozoan parasite that causes toxoplasmosis. It is capable of infecting all warm-blooded animals, including humans, where pregnant women and immunocompromised subjects are the main risk groups. The parasite can be transmitted transplacentally; it can cause blindness and mental defects in congenitally infected children or even fetal death [1].

The primary route of food-borne infection is the ingestion of undercooked or cured meat containing viable parasite cysts [2,3,4,5,6]. In the epidemiology of toxoplasmosis, pork plays a significant role, as pigs are highly susceptible to infection, even with a minimal infective dose of 1 oocyst [7]. Moreover, the European Food Safety Authority (EFSA) considers *Toxoplasma* one of the most significant biological risks in the context of pork inspection; currently, however, this parasite is not routinely controlled [8].

*Toxoplasma gondii* has a high affinity for neural and muscular tissues. The parasite invades, multiplies, and persists in several types of pig tissue, particularly the brain, heart, tongue, and diaphragm. Therefore, to obtain an indication of infection in edible tissues, sampling procedures should take those particular tissues into account [9,10]. However, as the number of *T. gondii* cysts in meat from infected food animals may be very low and unevenly distributed, the parasite is difficult and laborious to detect via direct methods [11,12]. The sensitivity of *T. gondii* detection can be increased by using molecular techniques in combination with bioassay [13].

Among the various meat products derived from pork, dry-cured ham stands out as a high-quality product of increasing economic relevance in the Mediterranean region. Spain is one of the leading producers and exporters. Curing has often been regarded as an effective technology against *T. gondii* [14,15]. Nevertheless, several studies have highlighted that curing may not completely inactivate all bradyzoite cysts [16,17,18]. Thus, to guarantee consumer health, pork legs intended for the elaboration of cured ham would normally need to be tested individually. However, this procedure is destructive, leading to economic loss; it would thus be useful to take target tissues of lower commercial value in the same animal as indicators of the parasite’s presence. Studies have been carried out on *T. gondii* in pig organs and commercial meat cuts [19,20,21,22]. However, to the best of our knowledge, little information is available on the association of contamination among them.

In view of that relationship’s importance for the meat industry risk assessment process, our study aimed to assess the presence of *T. gondii* in raw ham and target tissues (tongue and heart) of seropositive pigs and to use that information to select a representative testing tissue for the risk evaluation of pork meat destined for elaborating cured meat products. To increase the sensitivity of *T. gondii* detection, we additionally applied bioassays in mice and qPCR. This study complements the results of another previous study in our laboratory [18].

## 2. Materials and Methods

1. Sampling and sample preparation for analysis

Sampling of 38 naturally infected pigs is described elsewhere [18]. Three serum negative pigs (<1:20) were included. In addition to boneless raw hams, the heart and tongue from each pig were sampled, minced, and we prepared a target tissue pool by combining those two tissues at a 50% ratio. All resulting samples were immediately vacuum-packaged and stored under refrigeration (2 °C) until the time of analysis (<72 h).

2. Analysis of tissues for *T. gondii*

First, we applied a concentration technique with an acid pepsin digestion procedure to raw ham and the target tissues, as described elsewhere [23,24]. We then used aliquots of digestion extracts for analysis by mouse bioassay. When bioassay was negative, we tested for the presence of parasite DNA via qPCR. The number of positive results identified by either bioassay or qPCR yielded the total number of positive samples. Analytical procedures are described in Herrero et al. [18], and are summarized below.

### 2.1. Mouse Bioassay

Briefly, a 0.5 mL portion of the digest was injected intraperitoneally into eight 20–25 g female CD1 Swiss mice per sample. After 60 days blood samples were collected. Each bioassay included negative controls. Mice were housed at the Centro de Investigación Biomédica de Aragón (CIBA, Zaragoza, Spain). All procedures were authorized by the University of Zaragoza’s Biosecurity Commission and the Ethics Advisory Commission for Animal Experimentation (Report No. PI07/12), and consistent with international principles for animal research [25]. To detect antibodies against *T. gondii*, sera samples were analyzed by indirect immunofluorescence assay (IFA) (bioMérieux, Marcy l’Etoile, France) and examined with a fluorescence microscope (Eclipse 80i, Nikon instruments INC, Netherlands). A titer of ≥1:10 was considered positive, previously established in our laboratory [26]. A positive and a negative control serum were included in each test.

### 2.2. qPCR Analysis

Genomic DNA from *T. gondii* was extracted from 100 µL of digestion extract using the Ultra-Clean^®^ Tissue & Cells DNA Isolation Kit (Ref. 12334-S; MoBio Laboratories, Inc., Carlsbad, CA, USA), following the protocol supplied with the kit. Real-time PCR assays were then performed on a CFX Connect instrument (Bio-Rad Laboratories), targeting the 529 bp repetitive element and the SAG genes. Each sample was analyzed in triplicate and included a negative control, a positive control, and an additional reaction amplifying actin gene copies as an internal control. Quantification was based on the cycle threshold (Ct) value of the 529 bp repeat element while amplification of the SAG marker was used to confirm the result. Positivity was defined as Ct < 38, based on standardized protocol and conditions established in our laboratory. Parasite load in positive samples was further estimated using a calibration curve, characterized by a slope of 3.329. The curve showed a coefficient of determination (R^2^) of 0.992 and a reaction efficiency of 99.7%, all determined under standardized laboratory conditions. Standards with known parasite concentrations are included in every qPCR run as internal controls to validate the assay, ensure data reliability, and enable accurate quantification under consistent conditions.

3. Statistical analysis

Differences in *T. gondii* detection between raw hams and target tissues were assessed with Pearson’s Chi-square test, applying the Likelihood Ratio test when Pearson’s Chi-square test was not valid. Agreement between the test outcomes and the different tissues was evaluated using Cohen’s kappa coefficient (and its standard error). Statistical analyses were performed with IBM SPSS 29.0 for Windows. Differences were considered statistically significant when *p* < 0.050.

## 3. Results

IFA and qPCR findings from raw ham and target tissues (heart and tongue) from the 38 seropositive pigs analyzed are shown in Table 1. *Toxoplasma gondii* was detected in 26 raw hams and in 26 samples of target tissues (68.4%). Twenty-two out of twenty-six positive samples came from the same animals, whereas 4 samples were discordant cases.

The parasite was detected in 4 hams from pigs with a serological titer < 1:80 (28.6% (4/14)), and in 22 hams of pigs with a titer ≥ 1:80 (91.7% (22/24)). Five of the twelve ham samples with negative results in the bioassay were positive by qPCR, reaching Ct values ranging from 34.45±0.25 (corresponding to 118 parasites/g) to 36.89 ± 0.40 (23 parasites/g).

The parasite was detected in 3 target tissue samples corresponding to pigs with a serological titer < 1:80 (21.4% (3/14)), and in 23 target tissue samples of pigs with a titer ≥ 1:80 (95.8% (23/24)). Only one of the digestion extracts, which was bioassay-negative, resulted in a positive qPCR, with a Ct value of 36.84 ± 0.12 (24 parasites/g).

**Figure 1:**
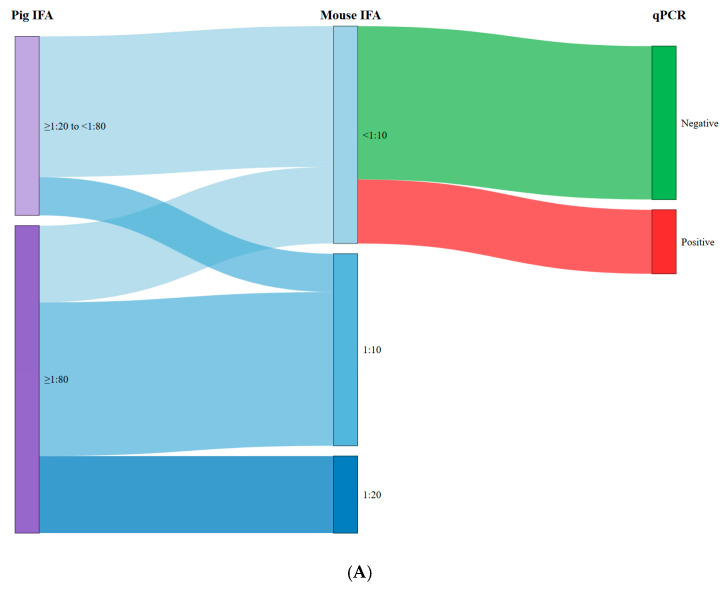

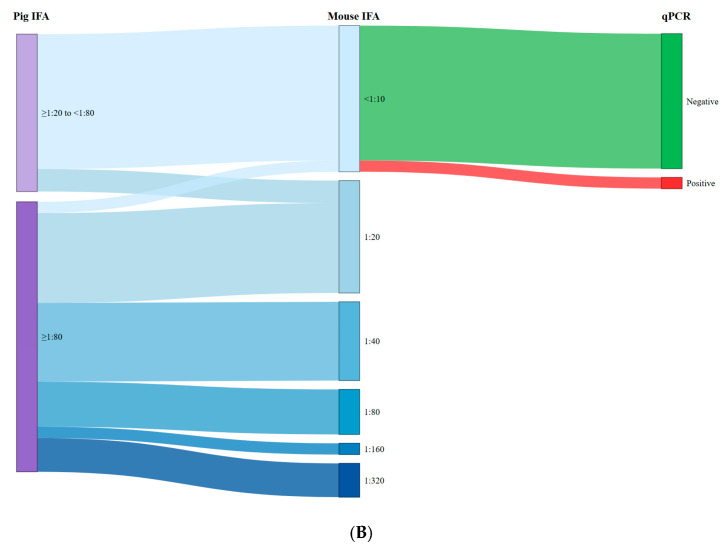
Relationship between the antibody titres of the pigs tested and the titres of mice inoculated with raw ham (**A**) and target tissues (**B**) and qPCR results.

Figure 1 shows Sankey diagrams illustrating the relationship between the antibody titers of the pigs tested and the titers of mice inoculated with raw ham (Figure 1A) and target tissues (Figure 1B), as well as with the qPCR results. As can be observed in Figure 1A, high antibody titers in pigs (≥1:80) were associated with seropositivity in mice following inoculation with extracts derived from raw ham samples (75.0% (18/24)). Conversely, seropositive pigs showing low titers (<1:80) resulted in a lower percentage of seropositive mice (21.4% (3/14)). A similar trend was observed for target tissues (Figure 1B), where the association between pigs with low titers and mice seronegativity (85.7% (12/14)) and pigs with high titers and mice seropositivity (95.8% (23/24)) was even more pronounced. In both raw ham and target tissues, we observed a strong association between mouse seronegativity and qPCR negativity, specifically 70.6% and 92.3%, respectively. Nevertheless, the antibody titers obtained in mice inoculated with target tissue samples were significantly higher (*p* < 0.001) compared with those inoculated with raw ham extracts. Specifically, the titer range for mice inoculated with raw ham was 1:10–1:20, compared to 1:20–1:320 for those inoculated with target tissues.

No significant differences (*p* > 0.050) were found in the results obtained from both fresh tissues (positive and negative samples). Our statistical analysis comparing results of ham and target tissue samples (as defined in 3. Materials and Methods) yielded a strong level of agreement (κ = 0.790 ± 0.100), indicating that the results were comparable (i.e., similar) (Table 2).

## 4. Discussion

Our study aimed to investigate the presence of *T. gondii* in raw ham and target tissues (heart and tongue) of seropositive pigs and to use that information to select a representative testing tissue for easy risk evaluation of pork destined for the elaboration of cured meat products.

Given that clinical manifestations of toxoplasmosis are generally absent in pigs and the detection of tissue cysts during routine *post-mortem* inspection at slaughter is virtually unfeasible, infected pigs often go unnoticed; their meat thus becomes a potential source of infection for consumers [8,27]. Furthermore, the detection of viable parasites isolated from cured meat products [17,18,28,29] highlights the efficacy of this technological hurdle in reducing but not in completely eliminating the risk.

Thus, to guarantee consumer health, it is essential to control fresh pork for *T. gondii*. For this reason, it is imperative to standardize detection methods in order to implement control measures [30]. However, the parasite’s detection in tissue samples is a technically demanding analytical procedure that requires optimization by selecting appropriate target organs. Tissue cysts are not homogeneously distributed across an animal’s organs: this may reduce detection sensitivity and consequently make results less reliable.

Dubey et al. [7] noted that the parasite, after infection, can be found in almost all swine tissues, particularly in the brain, skeletal muscles, and cardiac muscle. Muscle tissues were those that presented the lowest quantities of parasites. However, several researchers have used muscle tissue to detect *T. gondii* and have obtained good results in their analyses. Other authors, such as Juránková et al. [20], evaluated the most suitable tissues for parasite qPCR detection in pigs and identified the brain as the primary predilection site for *T. gondii*, exhibiting the highest number of positive samples and the lowest Ct values, indicative of the greatest parasite burden. Moreover, their findings showed that the heart and lungs harbored the second-highest levels of parasitic load. Similar results were reported by Gisbert Algaba et al. [21], who detected the presence of *T. gondii* in all the tissues they investigated (heart, brain, lungs, and the muscles *gastrocnemius, psoas major, longissimus dorsi*, diaphragm, and intercostals), confirming that heart and brain are the predilection tissues for the direct detection of the parasite.

The brain, although a highly reliable target organ, presents sampling difficulties for routine control. In this study, our aim was to take a realistic, industry-oriented approach by developing a practical control tool for the meat production sector while providing reliable data for risk assessment. To increase efficiency, it would thus be more advisable to resort to alternative tissues. In agreement with our results, Miura et al. [31] credited a high isolation rate (69.2%) in 400 pigs slaughtered for human consumption to the sampling of several different tissues (heart, diaphragm, liver, tongue, and masseter). In a study investigating the occurrence of *T. gondii* in pigs slaughtered in Brazil, Silva et al. [32] detected parasite DNA in all tissues under analysis (tongue, diaphragm, and masseter). Dámek et al. [28] investigated the presence and concentration of *T. gondii* DNA in various organs and muscles, including the shoulder, breast, ham, and heart of infected pigs. They detected a higher proportion of *T. gondii* DNA by MC-qPCR in heart (87.5%) compared to muscle samples (41.7%) and estimated that the number of parasites per gram of tissue was highest in the hearts and lowest in the hams.

Considering that hams are high-value cuts from the carcass, we thus regard heart and tongue samples as a good alternative model of edible meat tissue. They can be easily obtained and tested, particularly as there is strong agreement between them. Furthermore, we observed that the immune response in the bioassay mice was stronger for samples derived from target tissues, reaching titers of up to 1:320, whereas in ham the maximum values were 1:20. These results support the notion that the parasite burden tends to be higher in the target tissues we selected (heart and tongue) than in ham. These tissues can thus be used as reliable indicators for surveillance measures in the meat industry. Our results are in agreement with Dubey et al. [7], who previously showed that muscle tissues present comparatively low quantities of *T. gondii*.

To enhance the sensitivity of *T. gondii* detection, we combined two standardized techniques: quantitative PCR (qPCR) and mouse bioassay. Similarly, Burrells et al. [13] employed a molecular technique, magnetic capture qPCR (MC-qPCR), in combination with mouse bioassay, which is regarded as the gold standard method for isolating the parasite. The methodology of mouse bioassay is admittedly time-consuming and costly; it is thus inadequate for routine diagnosis but remains a valuable method for estimating the risk of *T. gondii*.

As previously mentioned, no specific control measures for *T. gondii* are currently in place for routine meat inspection. However, serology may help estimate the risk of human exposure, because *T. gondii* seroprevalence in farm animals closely reflects the likelihood of tissue cysts being present in their meat. The probability of detecting *T. gondii* parasites in seropositive animals has been demonstrated to be very high in pigs (58.8–95.8%) [10,26]. Nevertheless, seroprevalence in pigs does not always align with the presence of tissue cysts in pork [33]. As a result, more investigations are necessary to confirm an unequivocal association between *T. gondii* seropositivity in pigs and parasite detection in pork.

In conclusion, our findings confirm that pig seropositivity can serve as a useful indicator of *T. gondii* risk in pork when antibody titers are ≥1:80 (cut-off value), in spite of the small number of samples analyzed. Mouse bioassay and qPCR are complementary techniques that enhance the detection of parasites in pork. Moreover, the tongue and heart demonstrated a strong potential for detecting *T. gondii* by using this analytical methodology, although these tissues were not analyzed separately in this study. Therefore, we propose a risk management strategy for the cured-ham meat industry that involves analyzing these target tissues (heart and tongue) using bioassay combined with qPCR. This approach preserves the economic value of hams while ensuring a reliable and effective assessment of *T. gondii* risk.

## Figures and Tables

**Table 1:** Presence of *Toxoplasma gondii* in tissues from seropositive pigs.

Pig Titers	Analytical Technique	Raw Ham	Heart and Tongue
**<1:80 (n = 14)**	Bioassay ^a^	**3** (1:10)	**2** (1:20)
	qPCR ^b^	**1** (34.45 ± 0.25; 118)	**1** (36.84 ± 0.12; 24.0)
**≥1:80 (n = 24)**	Bioassay ^a^	**18** (1:10–1:20)	**23** (1:10–1:320)
	qPCR ^b^	**4** (36.00 ± 0.64; 42.7–36.89 ± 0.40; 23)	**0**
**Total of positive samples**		**26**	**26**

^a^ Bioassay: number of positive samples (mice titers). Number of dead mice during the bioassay: 28. ^b^ qPCR: number of positive samples (Ct values ± SD; parasites/g). Samples were only analyzed by qPCR if the bioassay was negative.

**Table 2:** Diagnostic agreement between type of tissues.

	Raw Ham Negative	Raw Ham Positive	Total
**Target tissue negative**	13	2	15
**Target tissue positive**	2	24	27
**Total**	15	26	41

## Data Availability

The original contributions presented in this study are included in the article. Further inquiries can be directed to the corresponding authors.
